# Properties and Skin Compatibility of Films Based on Poly(Lactic Acid) (PLA) Bionanocomposites Incorporating Chitin Nanofibrils (CN)

**DOI:** 10.3390/jfb11020021

**Published:** 2020-04-01

**Authors:** Maria-Beatrice Coltelli, Laura Aliotta, Alessandro Vannozzi, Pierfrancesco Morganti, Luca Panariello, Serena Danti, Simona Neri, Cristina Fernandez-Avila, Alessandra Fusco, Giovanna Donnarumma, Andrea Lazzeri

**Affiliations:** 1 Department of Civil and Industrial Engineering, University of Pisa, 56122 Pisa, Italy; laura.aliotta@dici.unipi.it (L.A.); alessandrovannozzi91@hotmail.it (A.V.); luca.panariello@ing.unipi.it (L.P.); serena.danti@unipi.it (S.D.); alessandra.fusco@unicampania.it (A.F.); giovanna.donnarumma@unicampania.it (G.D.); andrea.lazzeri@unipi.it (A.L.); 2 Consorzio Interuniversitario Nazionale per la Scienza e Tecnologia dei Materiali (INSTM), 50121 Florence, Italy; 3 Academy of History of Health Care Art, 00193 Rome, Italy; pierfrancesco.morganti@iscd.it; 4 IRIS Technology Solutions S.L, 08860 Castelldefels, Barcelona, Spain; sneri@iris.cat (S.N.); cfernandez@iris.cat (C.F.-A.); 5 Department of Experimental Medicine, University of Campania “Luigi Vanvitelli”, 80138 Naples, Italy

**Keywords:** poly(lactic acid), poly(butylene succinate), chitin nanofibrils, starch, skin compatibility, anti-microbial

## Abstract

Nanobiocomposites suitable for preparing skin compatible films by flat die extrusion were prepared by using plasticized poly(lactic acid) (PLA), poly(butylene succinate-co-adipate) (PBSA), and Chitin nanofibrils as functional filler. Chitin nanofibrils (CNs) were dispersed in the blends thanks to the preparation of pre-nanocomposites containing poly(ethylene glycol). Thanks to the use of a melt strength enhancer (Plastistrength) and calcium carbonate, the processability and thermal properties of bionanocomposites films containing CNs could be tuned in a wide range. Moreover, the resultant films were flexible and highly resistant. The addition of CNs in the presence of starch proved not advantageous because of an extensive chain scission resulting in low values of melt viscosity. The films containing CNs or CNs and calcium carbonate resulted biocompatible and enabled the production of cells defensins, acting as indirect anti-microbial. Nevertheless, tests made with *Staphylococcus aureus* and *Enterobacter* spp. (Gram positive and negative respectively) by the qualitative agar diffusion test did not show any direct anti-microbial activity of the films. The results are explained considering the morphology of the film and the different mechanisms of direct and indirect anti-microbial action generated by the nanobiocomposite based films.

## 1. Introduction

In the last decades, extensive academic and industrial research has been focused on the development of bio-based and biodegradable polymers with specific functional properties for decreasing the environmental impact of many general products, especially those with a very short life, and thus responsible for much of the concern related to environmental issues [1,2,3]. The production of biobased films with anti-microbial and anti-inflammatory properties that can be easily integrated in the current industrial processes for producing diapers or other pads can be thus extremely useful for decreasing the environmental impact of sanitary products. As an example, disposable baby diapers contribute globally about 77 million tons of solid waste to landfills, with a degradation period of at least 500 years [4]. Regarding the parts of the diaper in contact with skin, biobased and biodegradable versions with improved compatibility with skin would be a very good alternative for health reasons also. In fact, by combining biobased materials and functional biopolymers, it seems possible to limit cases of rash, dermatitis, and inflammation phenomena that are still present in the population using sanitary products. For instance, adding to films anti-microbial and/or anti-inflammatory substances can be a good strategy [5]. It is known that topical exposure to a variety of xenobiotics may result in irritant as well as allergic contact dermatitis in humans and that the cytokines play a pivotal role in immune and inflammatory reactions [6]. In this article, cytokine levels were used as a marker for skin sensitizer prediction. On the other hand, a direct anti-microbial activity can be attributed to substances that are toxic for bacteria cells, but sometimes these substances are toxic also for human cells. An indirect anti-microbial action is, on the contrary, due to substances stimulating skin cells vitality and inducing them to produce antimicrobial peptides (defensins) and can be anyway very desirable in sanitary applications. Moreover, thanks to this indirect approach, the problem of cytotoxicity is not relevant.

Chitin nanofibrils (CNs) possess good antimicrobial properties [7], but they also act as indirect anti-microbial, stimulating the immunomodulatory activity of skin cells [8,9]. Chitin nano-fibrils have an average size of 240 nm × 7 nm × 5 nm and their shape is like thin needles. They can be easily metabolized by the human body and promote the proliferation and adhesion of cells [9]. Consequently, they can be used in cosmetic and biomedical sectors [10], but their potential can also be exploited in sanitary and food packaging sectors [11].

For the production of sanitary films, aliphatic polyesters are the most promising materials. In fact, they are environmentally friendly and at the same time they offer good compromise of mechanical properties; furthermore, thanks to their biocompatibility, they were intensively investigated for applications in the biomedical sector [12,13]. Among the biodegradable polyesters, one of the most studied is the poly(lactic acid) (PLA). PLA is completely biobased, as it is obtained from the fermentation of corn starch, sugar beets or other renewable resources [14,15]. Regarding its properties, at room temperature it exhibits a Young’s modulus of about 3 GPa and a tensile strength between 50 and 70 MPa [16,17]. However, the low deformation at break (around 4%) and low impact properties limits its applications. To improve the flexibility of PLA as well its the processability, different research activities were focused on the addition of biocompatible and biodegradable plasticizers like acetyl tributyl citrate (ATBC), triethylene glycol, lactic acid oligomer (OLA) etc. [18,19,20].

Polymer blending is also a successful strategy to improve the drawbacks of PLA. Blending PLA with poly(butylene adipate-co-terephthalate) (PBAT) revealed significant improvements in both mechanical properties (in particular, flexibility and toughness) and processability [21,22,23]. Nevertheless, PBAT is currently not completely derived from renewable resources.

A very promising biodegradable and biobased polymer that can be blended to PLA with good results is poly(butylene succinate) (PBS), which is a semicrystalline polymer synthetized from butanediol and succinic acid (both are available from biobased renewable resources). The starting mechanical properties of PBS are quite promising. In fact, it has excellent biodegradability, good thermal properties and it is very easily processable [24]. Nevertheless, PLA/PBS blends have some limitations related to an insufficient ductility and stiffness. To overcome these limits, many attempts were done to improve the PLA/PBS blends properties via plasticization. To this purpose, in order to have a completely biobased product, the use of a suitable biobased plasticizer is of fundamental importance [25]. PLA/PBS blends plasticized with poly(ethylene) glycol (PEG) have been investigated and an increment in elongation at break and softness thanks to plasticization were reported [1,26,27]. The use of citrate derivatives such as tributyl citrate, acetyl triethyl citrate, and acetyl tributyl citrate (ATBC), have also been investigated in literature [28,29]. It was found that the addition of ATBC leads to flexibility and processability improvements in PLA blends. Furthermore, PLA/PBS/ATBC films, produced via flat-die extrusion, have shown an immunomodulatory behavior in tests performed with keratinocytes, suggesting a slight indirect anti-microbial effect [24]. The use of chain extenders has been revealed as fundamental in order to modulate the melt viscosity, thus improving the processability [30].

As a starting point, and on the basis of previous studies [25,31,32,33,34], in this work, a PLA/PBSA blend plasticized with ATBC is considered the matrix for producing nanobiocomposites containing CNs as functional filler.

The addition of CNs to PLA based blends can lead to a bioplastic nanocomposite material having improved mechanical and functional properties [35,36]. However, in order to reach good mechanical properties, an efficient dispersion of CNs is fundamental. In the literature, different research methodologies are reported for CN dispersion. A preliminary chemical modification of chitin nanofibrils was investigated by several authors [37,38,39,40], however the final mechanical properties of the material were not significantly improved. Furthermore, the preliminary chemical modification complicates the processing of the material and increases the final cost. It is essential to use an efficient process that can be easily transferable to an industrial scale considering that films are produced by the melt extrusion technique. In this sense, the production of pre-composite by using a suitable plasticizer can represent a good solution. The plasticizer has a synergistic effect: it improves the processability and flexibility and at the same time it can facilitate the dispersion of the CNs. Citrates plasticizers have been investigated in literature; thanks to their ester groups, they have a very good affinity with the PLA matrix and favor the CNs dispersion [41,42,43]. However, it has been demonstrated that another very effective plasticizer for the dispersion of CNs is the poly(ethylene glycol) (PEG) [32,34,44,45].

For its low cost, renewability, and high compatibility with CNs, starch can be another good filler that can be added with CNs in order to reduce the final material cost [45]. However, native starch is not chemically compatible with the PLA matrix, and if there is not a good dispersion of the starch granules [46,47], the resulting PLA composite material will be more brittle. A good technique that was investigated in the literature is starch granule plasticization, allowing a better dispersion of the starch granules. Different types of plasticizer have been widely studied such as water [48], urea [49], citric acid [50], glycerol [51], and polyethylene glycol (PEG) [52]. These plasticizers are capable of breaking the hydrogen bonds within the starch granules. However, a drawback that can occur using these plasticizers, is that they can accelerate the degradation of PLA by hydrolysis when they are processed in the molten state [46].

In this work, starting from a PLA/PBS/ATBC matrix, where the ratio between the various components was selected based on a previous work [25], bionanocomposites including CNs were prepared. The effect of a melt strength enhancer and contemporarily of CNs alone or mixed with starch was analyzed. In order to have a better dispersion of CNs, a master-batch preparation with PEG was carried out before the blend extrusion. PEG 6000, solid, was used for composites containing only CNs, while for composites also containing the native starch, the liquid PEG 400 was used for an easy pre-mixing with starch at room temperature.

The blends were characterized in terms of their processability through torque and melt fluidity measurements. Moreover, they were compared in terms of mechanical and thermal properties discussing the results of tensile tests and differential scanning calorimetry (DSC) investigations respectively. The blends showing the best processability and thermo-mechanical performances were characterized in terms of compatibility with keratinocytes and immunomodulatory behavior as well as anti-microbial activity based on agar diffusion method with the aim of investigating biocompatibility and direct and indirect anti-microbial properties. All the results were discussed to identify materials and methodologies to enlarge the use of functional bionanocomposites in specific widely diffused skin contact applications.

## 2. Materials and Methods

### 2.1. Materials

Poly(lactic) acid (PLA) was purchased from NatureWorks LLC (Minnetonka, MN, USA), trade name PLA2003D. This is a special grade of PLA for extrusion process. According to data sheet it contains about 4% of D-lactic acid units, melt flow index (MFI) of 6 g/10 min (210 °C, 2.16 kg), nominal average molar mass 200,000 g/mol and density of 1.24 g/cm^3^.

Poly(butylene succinate) (PBS) was purchased from Mitsubishi Chemical Corporation (Tokyo, Japan), trade name BioPBS FD92PM. It is a copolymer of succinic acid, adipic acid and 1,4-butandiol. It is a soft and flexible semi-crystalline polyester suitable for both blown and cast film extrusion having a melt flow index (MFI) of 4 g/10 min (190 °C, 2.16 kg) and a density of 1.24 g/cm^3^.

Acetyl Tributyl Citrate (ATBC) from Tecnosintesi S.p.A (Bergamo, Italy) was used as plasticizer. It is a biobased and biodegradable plasticizer obtained from the acetylation of tributylcitrate. It appears as a colorless liquid [density 1.05 g/cm^3^, molecular weight: 402.5 g/mol].

Two typologies of Poly(ethylene glycol) (PEG) purchased from Sigma-Aldrich (St. Louis, MO, USA) were used without any further purification. In particular, a liquid PEG, having a low molecular weight of 400 g/mol (PEG 400), and solid PEG, with a high molecular weight of 6000 g/mol (PEG 6000), were used. PEG 6000 is a colorless solid with a solubility in water of 50 mg/mL at 20 °C. PEG 400 is a liquid soluble in Toluene, Acetone and Ethanol, dispersible in water and with a density of 0.985 g/mL.

Plastistrength 550 (named PS for brevity) was purchased from Arkema (Paris, France). It is a medium molecular-weight acrylic copolymer that appears as a white powder (density: 1.17 g/cm^3^). It is a commercial processing aid added to improve the melt processability [53].

Wheat native starch is a white odorless powder, insoluble in water, commercialized by Sacchetto SPA (Cuneo, Italy). It is a dry product with a very low protein content of vegetable origin, with a humidity content < 13% and a pH (sol. 20% water dist.) between 5.0 and 7.0. It consists mainly of amylose, amylopectin and water with an ash content < 0.25%.

Chitin nano-fibrils (CN) water suspension (2 wt.% of concentration) were provided by MAVI SUD (Latina, Italy). These CNs are produced by means of a patented process starting from chitin coming from seafood waste [54]. Thanks to this process a stable water suspension, containing 300 billion of chitin nano-crystals for each millimeter, can be obtained. Furthermore, MAVI optimized the methodology necessary to produce these chitin nano-crystals industrially maintaining the specific biologic activity [31]. Consequently, CNs are perfect for those applications (like cosmetic) in which the skin-compatibility is an essential requirement. The CN water suspension was concentrated at 20% by weight for the pre-composites preparation [33].

Calcium carbonate was received from Omya SpA (Massa Carrara, Italy), trade name Omyacarb 2-AV. It is a white powder with a specific weight of 2.7 g/cm^3^ and a refractive index of 1.59. The product has a micrometric particle size, a diameter value relative to the maximum distribution curve (d 98%) of 15 µm, and 38% of particles having a diameter lower than 2 µm. The average statistical diameter (d 50%) is 2.6 µm.

### 2.2. Methods

#### 2.2.1. Brunauer–Emmett–Teller (BET) Characterization of Chitin Nano-Fibrils

A BET analysis of the chitin nano-fibrils was carried out, with a Micromeritics instrument -Gemini V analyzer (Micromeritics, Atlanta, GA, USA), in order to determine the surface area and the total porosity of chitin. The measurement was performed with nitrogen as measuring gas and helium for the calibration phase. Samples were conditioned at 120 °C for 6 h to remove the humidity. The degasification time was 30 min. The range of the ratio P/P_0_ (where P_0_ is the room pressure) for the analysis was between 0.05 and 0.3. The results of the measurements are the BET values that represent the surface area of the sample.

The morphology of chitin nanofibrils was investigated by a field emission scanning electron microscope (FESEM) FEI Quanta 450 FEG (FEI, Hillsboro, OR, USA). The water suspension, at 2 wt.% of chitin nanofibrils, was diluted 1:1000 and then one drop of the diluted suspension was deposited onto a glass window in order to make the FESEM analysis. At this point, from the SEM analysis on the chitin diluted sample, it was possible to obtain a theoretical area per gram of sample using the Image J software (NIH, Bethesda, MD, USA) (to calculate the average width and length of the nano-fibrils). Considering a chitin density value of 1.425 g/cm^3^ [55], the average mass of each fibril was obtained and the number of particles per gram was calculated by dividing a gram for the mass of a single fibril. Considering the fibrils as parallelepipeds, it was possible to evaluate the area and then a BET theoretical value by multiplying the area for the number of particles per gram.

#### 2.2.2. Chitin Master-Batch Preparation

To better disperse and to avoid the agglomeration of chitin nanofibrils, a dispersion with PEG was prepared. On the basis of a previous study it was observed that PEG can be intercalated between the CNs and it avoids the formation of compact CNs agglomerates [32].

Two pre-composite master batches were prepared with a high pre-dispersed chitin nano-fibrils content. The first one (named MB1) contained 50% of chitin nano-fibrils and 50% of PEG6000. The second one (named MB2) was prepared at 80 °C with chitin nano-fibrils, PEG400 and plasticized starch at 2:2:3 ratio. In both cases, the additives were added to the stirred chitin suspension and the drying was done in a rotavapor (Buchi R-210, buchi, Flawil, Switzerland) equipment at 50 °C and 100 mbar to avoid chitin degradation.

PEG 6000 in the MB1 was chosen in order to obtain a solid powder that can be easily fed during the extrusion process; at this purpose the use of PEG 6000 is fundamental instead of PEG 400 (that have a lower molecular weight). On the other hand, the use of liquid PEG 400 is essential for the preparation of MB2 due to the use of starch in powder form.

#### 2.2.3. Blends Preparation

Composites containing CNs and starch were obtained using an HAAKE Minilab II twin-screw mini-compounder (HAAKE, Vreden, Germany). This equipment is able not only to compound the molten material, but at the same time it is able to make torque measurements of the molten material.

For selecting the processing conditions, the thermal stability of the different additives were considered [9,25,51,56]. Moreover, it was taken into account that the thermal degradation of PLA/PBS blends occurs above 340 °C [57].

Before the extrusion, the polymers granules were dried in an oven at 60 °C for 24 h. In order to record the torque values for all the formulations, for each extrusion compounding 6 g of PLA/PBS pellets were manually mixed together with the other additives. The mixture was fed into the co-rotating mini-extruder with the help of a suitable little hopper. The processing temperature was set at 190 °C and the screws rotating speed was 110 rpm. After the introduction of the material, the molten material, pushed by the screws, was flushed in a back-flow channel (with the exit valve of the die closed) for 1 min. During this period, the torque value was recorded as a function of time. The extruded material was recovered (opening the die valve) after one minute of rotation inside the mini-extruder chamber to ensure a correct mixing.

At least ten experimental torque measurements were carried out for each blend to assure the reliability and consistency of the test and also to recover enough material (about 60 g per blend) for further tests. The final torque value represents the most significant value for the sample as the melt stabilizes. All the extrusion process, including the feeding operations, had a duration of 120 s to avoid degradation phenomena.

#### 2.2.4. Melt Flow Rate

The melt flow behavior of the blends was investigated with a Melt Flow Tester M20 (CEAST, Torino, Italy) equipped with an encoder. The instrument is able to measure the melt volume rate (MVR) of the polymer blend acquired by the encoder that follows the movement of the piston. Before the test, granules of the blends obtained from the mini-extruder were dried in an oven (set at 60 °C) for one day.

The melt flow rate (MFR) is defined as the weight of the polymer, at the molten state, that passes through a capillary (having a specific length and diameter) in 10 min under pressure applied by a specific weight (according the ISO 1133:2005). For this work the standard used was the ISO 1133D custom TTT where the following procedure was adopted: the sample was preheated without the weight for 40 s at 190 °C, then the weight of 2.160 kg was released on the piston and after 5 s a blade cut the spindle starting the real test. At this point the MVR was recorded, every 3 s, by the encoder.

All the MVR data were reported with their standard deviation thanks to the CEAST Visuamelt software of the equipment. The MFR values standard deviations were calculated by considering the results obtained by the measurements.

#### 2.2.5. Mechanical Testing

The mechanical characterization of the blends was carried out on films prepared by compression molding. At this purpose, the recovered material coming out from the mini-extruder (having the die rectangular die section) was manually pelletized. Before the compression molding the pellets were kept in a circulating air oven at 60 °C for 24 h to avoid water uptake. Different film for each formulation were prepared in a NOSELAB ATS manual laboratory heat press (Noselab, Milano, Italy). About 5 g of granules were put between two Teflon sheets and then pressed at 180 °C for 1 min with a pressure of 3 tons. The samples for tensile tests were obtained from the film using an Elastocon cutting die (Elastocon, Brämhult, Sweden), into dump-bell shaped tensile specimens (ISO 527-2 type A).

Tensile tests were carried out on an INSTRON universal testing machine 5500R (INSTRON, Buckinghamshire, UK), interfaced with a MERLIN software (version 4.42, INSTRON, Buckinghamshire, UK) and equipped with a 100 N load cell. Compressed air grips were used and the initial grip separation was 25 mm while the crosshead speed was set at 100 mm/min. At least ten specimens were tested for each blend and the average values were reported.

#### 2.2.6. Differential Scanning Calorimetry

To perform the DSC characterization of selected formulations, a Q200-TA Instrument differential scanning calorimeter (DSC, TA Instruments, New Castle, DE, USA) equipped with a RSC cooling system was used with nitrogen flow set at 50 mL/min, as purge gas. Indium was adopted as a standard for temperature and enthalpy calibration of the instrument.

The thermal program was set according to the following procedure: the sample was heated, at 10 °C/min, to 220 °C where was held for 2 min (in order to delete the thermal history of the sample). Subsequently, the sample was cooled again to −40 °C at 10 °C/min and then reheated again up to 200 °C.

Melting temperature (T_m_) and cold crystallization temperature (T_cc_) of the blend were determined by considering the maximum of the melting peaks and at the minimum of the cold crystallization peak respectively. As a consequence, the enthalpies of melting and of the cold crystallization were determined from the corresponding peak areas in the thermograms. The crystallinity percentage of PLA was evaluated according the following equation:

(1) $$X_{c}=\frac{\Delta H_{m,PLA}-\Delta H_{cc,PLA}}{\Delta {H^{{}^{\circ}}}_{m,PLA}\cdot X_{PLA}}\;,$$

where Δ*H_m,PLA_* and Δ*H_c,PLA_* are the melting enthalpy and the enthalpy of cold crystallization of PLA, while *X_PLA_* is the weight fraction of PLA in the selected formulation. Δ*H°_m_,_PLA_* is the melting enthalpy of the 100% crystalline PLA (considered 93 J/g [58]).

#### 2.2.7. Skin Compatibility Tests

HaCaT cells are adherent cells often utilized in skin test for their high capacity to differentiate and proliferate in vitro [59]. Immortalized human keratinocyte HaCaT cell line (purchased from CLS–Cell Lines Service, Eppelheim, Germany), were cultured in Dulbecco’s Modified Eagle Medium (DMEM) supplemented with 1% Penstrep, 1% glutamine and 10% fetal calf serum (Invitrogen, Carlsbad CA, USA) at 37 °C in air and 5% CO_2_. The HaCaT cells, seeded in 12-well plates until 80% of confluence, were incubated for 24 h with the films F4 and F7. F4 and F7 films were sterilized by washing with ethanol before the test. At the end of this time, resazurine was added to the concentration of 0.5 mg/mL and incubated for 4 h.

#### 2.2.8. Evaluation of Inflammatory and Indirect Antimicrobial Properties

The cells, cultured as described above, were seeded inside 12-well TC plates until 80% of confluence, and incubated for 24 h with the F4 and for 6 h and 24 h with the F7 (n = 3). F4 and F7 films were sterilized by washing with ethanol before the test. At these endpoints, total RNA was isolated with TRIzol and 1 µm of RNA was reverse-transcribed into complementary DNA (cDNA) using random hexamer primers, at 42 °C for 45 min, according to the manufacturer’s instructions. Real time polymer chain reaction (PCR) was carried out with the LC Fast Start DNA Master SYBR Green kit using 2 µL of cDNA, corresponding to 10 ng of total RNA in a 20 µL final volume, 3 mM MgCl_2_ and 0.5 µM sense and antisense primers (Table 1). Real-Time PCR was used to evaluate the expression of interleukins TNF-α, TGF-β, IL-6, IL-8, IL-1α, IL 1β and antimicrobial peptide HBD-2.

#### 2.2.9. Anti-Microbial Tests

The antimicrobial capacity of the samples was assayed in vitro by the qualitative agar diffusion test. This method consists in the determination of the spectrum of action of the sample, according to resistance of the selected microorganisms. In this case, as an exploratory approximation, the test was performed against two typical bacterial colonizers of the normal human skin: *Staphylococcus aureus* (ATCC9144) and *Enterobacter* spp. (ATCC13047) (Gram positive and negative respectively). All test strains were obtained from American Type Culture Collection (ATCC).

Briefly, aliquots of 100 μL microbial suspensions adjusted to 5 × 10^5^ CFU/mL were added to a melted soft agar solution which was poured onto the surface of solid agar plate (soft-agar overlay method). Microbial suspension densities were previously quantified by the common plate count method in the appropriate culture conditions.

Samples were cut into disks with a diameter of 6 mm and sterilized by UV irradiation for 15 min. Also, sterile filter disks of same size were used as controls. Afterwards, disks were placed on the previously inoculated agar plates (4–5 disks per plate) and incubated at 37 °C for 24 h. After the incubation time, the presence of clear zones of growth inhibition was determined visually and the diameter of the inhibition halos was measured with a caliper. The assay was performed in triplicate for each microorganism.

The microorganisms were declared sensitive in the agar diffusion test against bacteria, when an inhibition zone of ≥10 mm was observed, indicating a positive result of the test, while as negative when no inhibition zone was present.

## 3. Results

### 3.1. Comparison between Experimental and Theoretical Chitin BET Values

**Figure 1:**
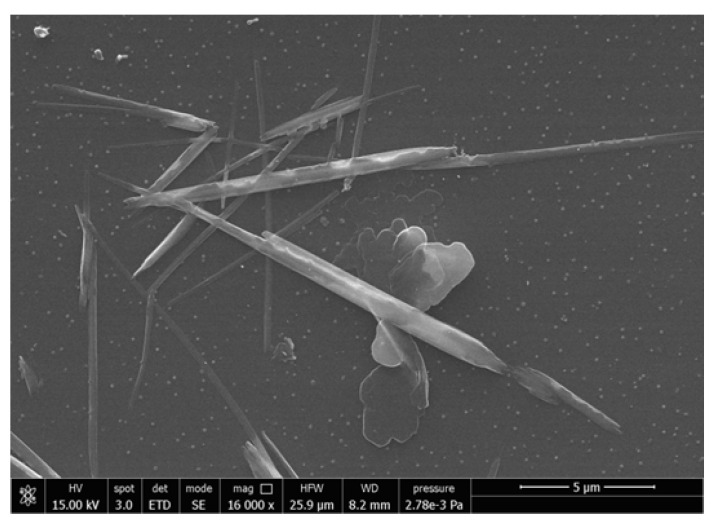
SEM image of a diluted 1:1000 sample of chitin suspension.

In Figure 1, the morphology of chitin nanofibrils is shown. By using image J software and following the procedure described in Section 4, the theoretical BET value, indicating the specific surface of the sample, was calculated and compared with the experimental one, measured for a sample obtained by drying up to constant weight the suspension of chitin nanofibrils. For the calculation of the average fibrils volume, a thickness of 5 nm was supposed based on what is reported in the literature [60]. The results are shown in Table 2.

From the data reported in Table 2, it is possible to observe a consistent discrepancy between the theoretical and the experimental BET values. This discrepancy can be ascribed to the formation of agglomerates because of the inter-fibrils interactions during the drying.

A sort of agglomeration factor (that will be named “weldability index” (WI)), capable to consider the percentage of agglomerated fibrils, was evaluated according to the following equation:

(2) $$\mathrm{WI}=\frac{\mathrm{theoretical}\;\mathrm{BET}-\mathrm{experimental}\;\mathrm{BET}}{\mathrm{theoretical}\;\mathrm{BET}}\times 100$$

A WI of 80% was obtained. The higher the weldability (WI) is, the more the particles are agglomerated providing a low experimental BET value. The result obtained supports that it is necessary to avoid this agglomeration by preparing a pre-nanocomposite with a plasticizer (PEG in this case) used as dispersant to maintain separated the nanofibrils in accordance with other literature results [32,42]. Moreover, based on the work of Pereira et al. [54], in the slightly acidic water suspension of CNs, a positive zeta potential is present. PEG, being a non-ionic surfactant, does not alter the full positive charge of CNs, but decreases its density, allowing a better dispersion in the more hydrophobic polyester blend. In good agreement it was demonstrated that this solid pre-nanocomposite, containing 50% by weight of chitin nanofibrils, can be then dispersed in the PLA based material [31,32].

### 3.2. Melt Properties

Different PLA/PBS formulations (Table 3) were prepared by extrusion to obtain biobased, biocompatible and flexible films. The ATBC was selected as plasticizer to increase the mechanical flexibility of the final material [7,48]. The melt strength enhancer Plastistrength (PS), based on acrylic copolymers, was also added up to 2 wt.% to modulate the viscosity during the processing of the blends containing chitin nanofibrils [25]. On the basis of the literature in this field, the CNs, added preparing pre-composites, and starch quantities were fixed to 2 wt.% and 3 wt.% respectively [52]. The F1 formulation, considered as a reference, consisted of only PLA, PBS, and ATBC. Their composition was selected considering the promising results of a previous work [25]. To better understand the effect of the Plastistrength (PS) addition, the F2 blend was prepared. Subsequently, the F3–F6 formulations were obtained replacing 2 wt.% of ATBC with PEG, having a similar plasticizing effect. Chitin nanofibrils were added only at 2 wt.% because, as they are dispersed at nanometric scale, low percentages are enough to obtain a homogenous distribution [32]. The addition of starch in the F5 and F6 blend was also evaluated as it is an additive acting as chitin nanofibrils carrier, because of its good compatibility with chitin, renewability, biodegradability and low cost. The F7 blend has the same composition of F4 but with the addition of micrometric calcium carbonate. The CaCO_3_ is used both as a slip agent and as a viscosity regulator during the processing.

The results of MFR and Torque for each blend are reported in Table 4. It can be observed that the addition of the melt strength enhancer (PS) to the starting formulation (F1), provokes an increment of torque (indirectly connected to the increase in melt viscosity) and consequently, a decrease in the MFR value. This result is in accordance with previous results [24] indicating that the viscosity increase due to PS addition is attributed to a combination of high miscibility and interactions between PLA and the acrylic copolymer-based products.

### 3.3. Tensile Properties

The results of the main mechanical properties (stress at break, elongation at break, and stress at yielding) are reported in Table 5.

It is evident that the addition of PS does not alter significantly the elongation at break and the stress at break of the starting blend based on PLA/PBS/ATBC. A slight increment of the stress at break followed by a decrement of the elongation at break is registered for the F2 blend in which the PS was added. However the addition of PS led to an increase in the yield stress compared to F1 that exhibits a behavior quite similar to an elastomer [25]. The addition of chitin alone (F3) or combined with PS (F4) induced a slight decrease in the elongation and stress at break. Both stress and elongation at break are lower than the F1 and F2 mixtures. Nevertheless, the yield stress of the F4 mixture is improved if compared to F2 mixture. The addition of chitin combined with PS led to an improvement of the final mechanical properties. The partial decrement of elongation at break and tensile stress at break is balanced by the increment of yield stress which provides to the final material an improved resistance in the elastic field. The final mechanical properties of the F4 blend are typical of flexible and ductile films and the CNs presence makes the use of this blend interesting for biomedical or cosmetic applications. The potential anti-microbicity and biocompatibility of these films can thus allow several new applications.

Contrary to what is reported in the literature [60], a decrement in mechanical performances was observed with the starch addition. Probably, during the processing, the starch carries a quantity of water higher than chitin and is not crystalline, so its hydroxyl groups have a stronger nucleophilic action. Consequently, the blends with starch, having a major quantity of humidity, lead to an inevitable matrix degradation (confirmed also by the MFR and Torque values) that worsens the final mechanical properties of the material. The degradation leads to shorter polymeric chains. Therefore, the addition of PS becomes useless because the interactions between macromolecules generated by PS are not sufficient for determining an effect similar to a physical cross-linking. This explains why no significant differences can be observed between F5 and F6 mixtures where the only difference is the PS addition.

The addition of CaCO_3_ to F4 formulation (F7 blend) led to a good balance of mechanical properties and at the same time it is possible to obtain a formulation with a good processability. The CaCO_3_ micro-particles are rigid fillers and decrease the elongation at break, but increasing the stress at yielding. However, it can be observed from Table 5 that the final mechanical properties of both CNs and Calcium Carbonate particles, probably due to their aggregation [61], causes a decrement of both elongation at break and yield stress.

### 3.4. DSC Characterization

The thermal characterization, by DSC analysis, was carried out on the best formulations selected by considering the mechanical tests. The F4 formulation showed the best performances, thus its thermal characteristics were compared with the starting blends (F1 and F2) and with the blend containing CaCO_3_ (F7).

**Figure 2:**
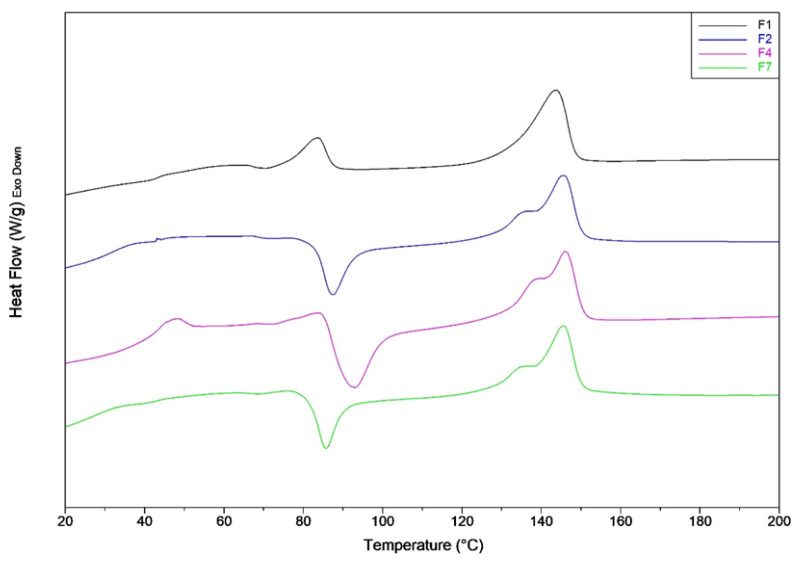
DSC first heating thermograms of F1, F2, F3 and F4 mixtures.

The results of the first heating scan, are reported in Table 6 and the relative thermograms in Figure 2. The first heating scan reveals the thermal history of the samples that are rapidly cooled after compression molding. It can be observed that there is not a significant change in the melting temperature for all the analyzed mixtures. Despite the rapid cooling, the F1 and F2 mixtures possess a significant crystalline fraction. Nevertheless, it can be observed that a net decrement of crystalline fraction is registered when PS is added (F2 blend). The result is in accordance to what is found in literature; in fact, the addition of acrylic copolymer-based product like PS, depresses the PLA crystal formation [25,62]. The addition of CNs provokes a significant further decrement of PLA crystallinity, thus the polymer remains almost amorphous.

In the presence of PS and also CNs (F2 and F4 blends), the exothermic crystallization peak is shifted to lower temperature. Thus, the presence of these additives favors the cold crystallization of PLA during the heating process. For the F1 mixture, the cold crystallization peak is almost negligible as it has a very small area coincident with a low value of cold crystallization enthalpy (3.05 J/g).

An enthalpic relaxation peak above the glass transition temperature due to the aging [63] is present markedly for F4 blend. Consequently, it can be deducted that the addition of CNs favors the PLA aging.

The CaCO_3_ addition, causes a marked increment of PLA crystallinity that passes from 2% for F4 blend to 13%. This result is in agreement with what can be found in the literature. In fact, it is known that CaCO_3_ acts as a heterogeneous nucleation enhancer for PLA, favoring and accelerating the crystallization process [64,65].

**Figure 3:**
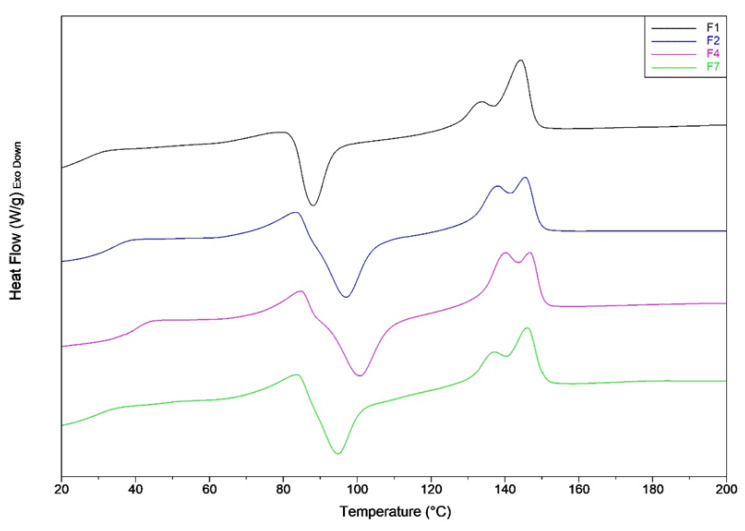
DSC second heating thermograms of F1, F2, F3 and F4 mixtures.

The results of the second heating scan are reported in Table 7 and Figure 3. Thanks to the plasticization of PLA, the glass transition temperatures of all mixtures are moved towards lower values (if compared with the glass transition temperatures of the first heating).

The PLA plasticization coupled with the annulment of thermal history, led to mixtures with a lower crystallinity content. In particular, the addition of the PS melt enhancer, allows to the samples to crystallize only during the heating step (not during the cooling), in agreement with a lower value of the crystallinity content in the sample. In fact, it can be observed that, for the second heating scan, the cold crystallization peak temperatures for F2 and F4 blends, as well as the crystalline content are similar. Probably, the crystal growth during cooling is blocked by intermolecular interactions that occur between PS and PLA.

### 3.5. Morphology of the Composites

**Figure 4:**
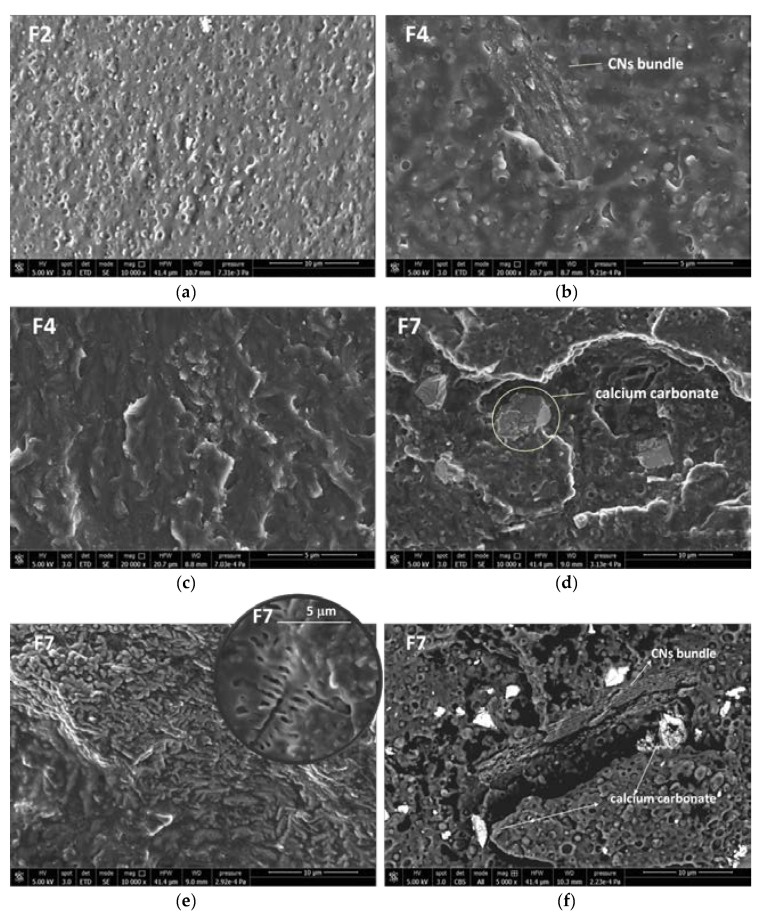
SEM micrograph related to: (**a**) F2; (**b**,**c**) F4; (**d**–**f**) F7. The micrographs (**a**–**e**) were obtained by the signal of secondary electrons (SEI modality). Micrograph (**f**) was obtained by signal due to backscattered electrons (CBS modality).

The plasticized PLA/PBSA blend containing 2% of PS showed the morphology represented by the micrograph of Figure 4a, where the spherical plasticized PBSA domains can be easily seen in the plasticized PLA matrix. By adding the CNs in the PEG masterbatch (Blends F4), the morphology of the bionanocomposites also results as in Figure 4b, but some additional CNs bundles can be observed.

The general morphology was homogenous, with CNs present inside the matrix as extended and well compatible bundles in all the nanocomposite (Figure 4c). When the calcium carbonate was also added the micrometric CaCO_3_ particles can be observed (Figure 4d) in the PLA/PBSA blend. Interestingly, in this blend some very extended regular structures are present with the shape typical of CNs bundles. Inside these bundles it can be observed a regular structure with a branched geometry (Figure 4e) having a shape and dimensions similar to the one shown by crystals of chitin nanofibrils in a previous work [7]. Hence these peculiar shapes can allow to observe the quite extended structure of CNs in the nanobiocomposite. By obtaining micrographs by backscattered electrons, it was possible to identify better the calcium carbonate as lighter particles, because of the higher atomic number of Calcium with respect to the other atoms in the blend. This kind of analysis evidenced that calcium carbonate was dispersed quite homogeneously in the F7 bionanocomposite, with some particles resulting close to CNs bundles (Figure 4f).

### 3.6. Skin Compatibility Results

The assessment of cell viability was performed by Alamar Blue assay in order to confirm the biocompatibility of the films. As shown in Table 8, F4 and F7 were not cytotoxic, indeed they promote, after 24 h of incubation, keratinocytes proliferation.

### 3.7. Evaluation of Antinflammatory and Indirect Antimicrobial Properties

Being inflammatory response a key factor in inducing skin sensitization, after 6 and 24 h of incubation Real-Time PCR was performed to evaluate the expression levels of pro- and anti-inflammatory cytokines produced by HaCaT cells treated with F4 and F7. In addition, the indirect antimicrobial activity of the films was examined by evaluating the expression levels of antimicrobial peptide HBD-2.

**Figure 5:**
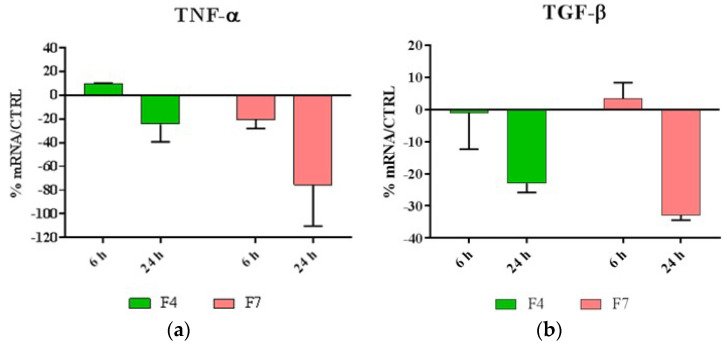

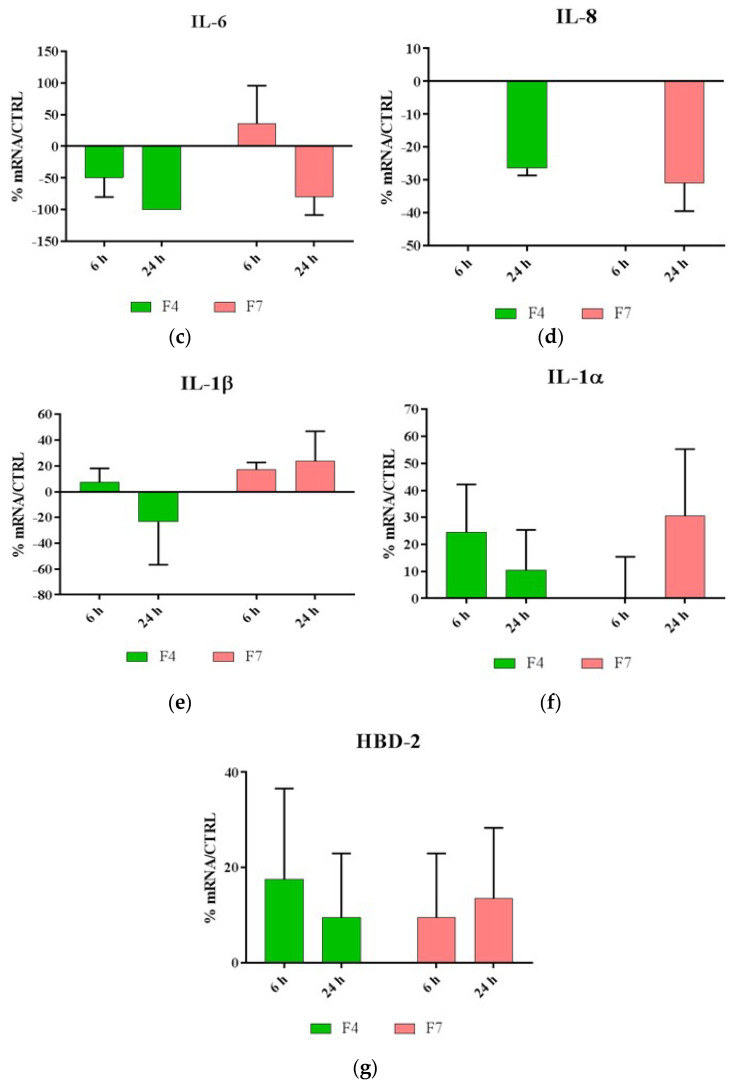
Relative gene expression of (**a**)TNF-α, (**b**)TGF-β, (**c**) IL-6, (**d**) IL-8, (**e**) IL-1α, (**f**) IL-1β and (**g**) HBD-2 in HaCat cells treated with F4 and F7 for 6 and 24 h. Data are mean ± SD and are expressed as percentage of increment relative to untreated cells (ctrl).

As shown in Figure 5, our results indicated that both F4 and F7 were able to reduce the expression of most proinflammatory cytokines, with the exception of IL-1, and were also able to strongly upregulate the expression of HBD-2.

### 3.8. Anti-Microbial Tests

**Figure 6:**
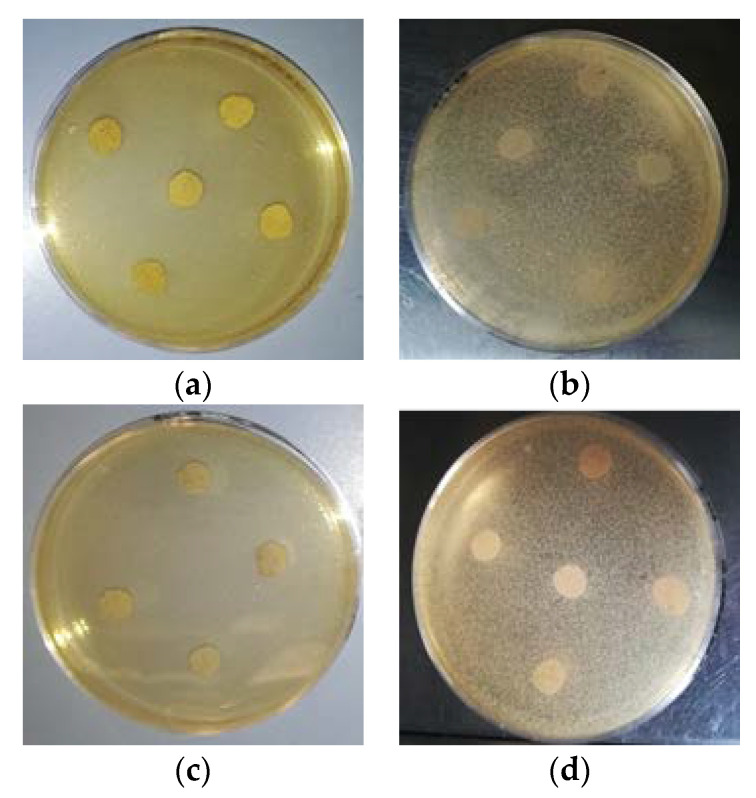
Plates of Agar diffusion test against (**a**,**c**) *S. aureus* and (**b**,**d**) *Enterobacter* spp., with no inhibition halos observed (inhibition zone ≤ 10 mm) for any sample tested: F4, F13 or F14.

Results of the agar diffusion test of the two PLA blend films formulations (F4 and F7) towards both Gram positive and negative bacteria reveal no effect. Hence, the films did not show any anti-microbial activity against these two bacteria (Figure 6).

F4 and F7 contained 2% (*w*/*w*) of dispersed CNs. This concentration was selected for its potential effect of reducing the growth of the bacteria strains tested, as observed in similar applications of the same CNs regarding food packaging [7]. Therefore, the lack of antimicrobial activity observed could be attributable to many factors, e.g., the low concentration of CNs distributed along the surface of the samples, since CNs are mainly embedded in the plasticized blend matrix.

## 4. Discussion

It can be observed that, compared to the F2 blend, the addition of chitin nanofibrils to PLA-PBS blend caused a slight decrease in melt viscosity. This behavior can be caused by the hydrolytic degradation of the polyester matrix caused by a not perfect drying of the CN/PEG pre-composite. In fact, the chitin nanofibrils are very hygroscopic, and the complete elimination of humidity is not an easy process. So, this torque decrement can be attributed to humidity traces that contribute to decrease the viscosity by shortening the polymeric PLA chains because of chain scission.

Nevertheless, the MFR and MVR, slightly decreased when the CNs were added, comparing F1 and F3. This effect, relevant in conditions of higher shear stress typical of the MFR test, also indicates the presence of good interactions between CNs, behaving as reinforcing agent, and the polymeric matrix.

**Figure 7:**
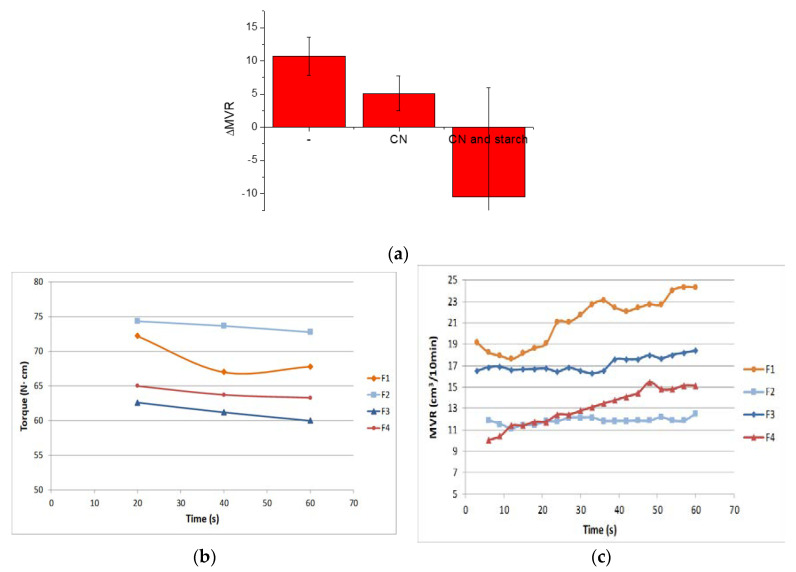
(**a**) MVR variation due to PS addition; (**b**) MVR trends as a function of time for F1, F2, F3 and F4 formulations; (**c**) Torque trends as a function of time for F1, F2, F3 and F4 formulations.

The variation of MVR due to the addition of the melt strength enhancer is reported in Figure 7a, and evidences very well that, in the presence of chitin nanofibrils, the melt strength enhancer is significant, but it seems less effective due to a more extensive chain scission. The strategy of regulating the fluidity of the melt by using PS is effective for controlling and tuning the nanobiocomposite processability.

In the samples where the starch was present, the difference is not significant at all. In these conditions the interactions occurring between PLA and PS are not effective in counterbalancing the very extensive chain scission. In the blends containing starch the torque resulted strongly decreased with respect to F1 and F2 because of extensive chain scission of the biopolyesters (Table 4).

In good agreement, the addition of starch (F5 and F6 blends) influenced in a significant way the MFR values. For F5 and F6 blend that contain starch, it was necessary to modify the test procedure because the mixtures were so fluid that the chamber emptied quickly not allowing the MVR measurement. For these mixtures the measurement time was accelerated (10 s instead of 1 min). From the results obtained it can be deducted that the addition of very small amount of pre-gelatinized starch (3 wt.%), are sufficient to cause an extensive degradation of both F5 and F6 mixtures even with the PS addition.

The CaCO_3_ addition to the F4 blend causes, as expected, a viscosity incrase that is reflected into a Torque increment and a lower MFR value.

Regarding the viscosity variation as a function of time during processing, in the case of extrusion lasting only 60 s, it was almost negligible (Figure 7b). Regarding the MVR as a function of time trends, as evidenced in Figure 7c, the reduction of slope observed between the F1 and F2 blend, suggesting a better stability of the F2 melt after the addition of PS, was not obtained comparing F4 and F3. The presence of CNs is thus making more complex the general processing behavior of the composites as a function of time. However, these variations, being slight, can be tuned and well controlled without compromising the general processability of the blends.

In Figure 7b,c, the MVR and Torque trends are reported as a function of time.

**Figure 8:**
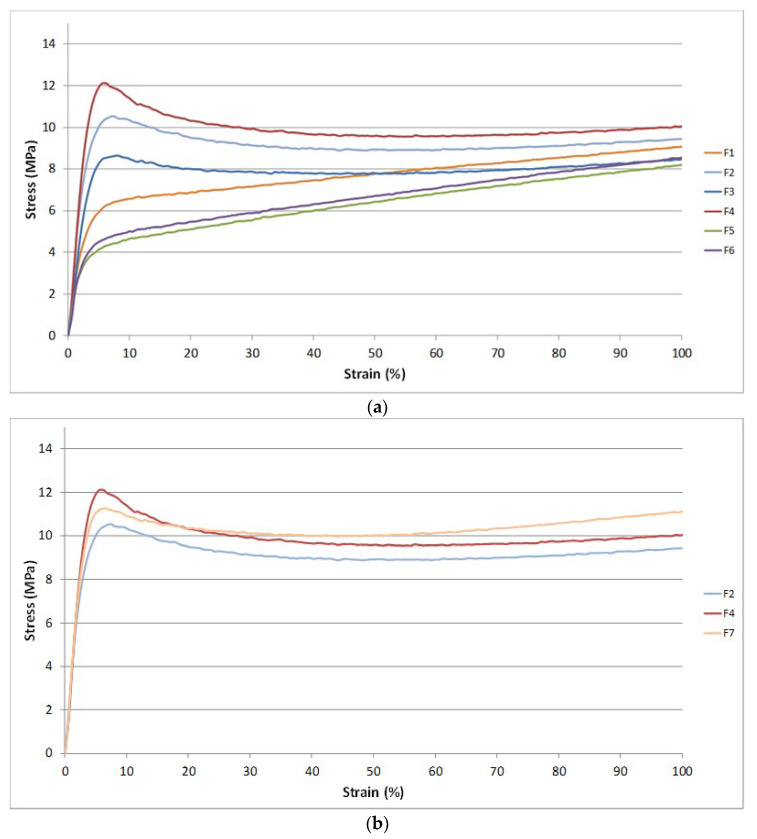
(**a**) First part of the stress-strain curves for the formulations without calcium carbonate; (**b**) Figure 2. F4 and F7.

Regarding the properties of blends films, In Figure 8a the first part of the stress-strain curves (up to 100% of elongation) for F1 up to F6 blends are reported. The addition of PS provoked an increase of yielding stress in both F2 and in F4 blends.

It can be noticed the improvement of the yield stress for the F4 mixture containing PS and CNs. However, also the CNs determined a change on the stress-strain curve shape. The CNs act like fillers increasing the yielding stress value due to significant interactions with the polymer matrix also thanks to orientation along the solicitation axis. The yielding is the point corresponding to the formation of neck in the specimens. More energy is required for necking if the interactions between macromolecules are more intense. Interestingly, the starch allowed to obtain stress-strain curves with a behavior more similar to an elastomer. The action of starch is thus different. In the first part of the stress-strain curve, its action completely deletes the reinforcing effect of CNs and seems to enhance the plasticization action of ATBC. In a similar way, but with a minor effect, the CaCO_3_ determines a decrease in stress at yield with respect to F4 (Figure 8b). These results evidence different mechanisms of interaction between the different fillers and the biopolyester chains during tensile tests.

Regarding thermal properties, CNs tend to hinder the PLA crystallization and the combination of CNs and PS showed a similar effect. This can be ascribed to the inhibition of crystal growth during cooling due to intermolecular interactions between PS and PLA.

The enthalpic relaxation peak above the glass transition temperature due to aging observed for F4 blend indicated that the addition of CNs favors the PLA aging. This result can be attributed to the orientation and alignment of macromolecules along the CNs surfaces, that favor the slow formation of ordered regions in the material. This effect is present in F4 but not in F7. This is due to the less homogeneous distribution obtained for CNs in the presence of calcium carbonate. In fact, the presence of fillers with different shapes, makes more difficult the macromolecular orientation. The complexity is also in agreement with the morphology analysis carried out by SEM. The PBSA formed a spherical dispersed phase in the plasticized PLA matrix and the CNs dispersion resulted homogeneous despite of the presence of complex bundles highly compatible with the matrix could be revealed in the blends. The addition of CaCO_3_ showed that both the fillers were well dispersed in the matrix where CNs bundles could crystallize, and interactions in between the two fillers could be also reasonably hypothesized.

The biocompatibility tests evidenced that both F4 and F7 films promoted cell proliferation. Moreover, they possess a significant immunomodulatory activity, in fact they are capable to downregulate the expression of IL, 6, IL-8, TGF-beta and TNF-alpha, and to upregulate the expression of IL-1 alpha and beta and HBD-2.

One of the functions of IL-1 in the epidermis is to promote keratinocyte differentiation, so we can hypothesize that F4 and F7 can stimulate cell differentiation while preserving an anti-inflammatory and antibacterial indirect activity.

It should be noted that the antibacterial activity occurs, in our experimental model, following the contact of the films with keratinocytes; this contact causes the induction of the production of defensins, antimicrobial peptides produced by epithelial cells. Hence, only when the film will be in contact with skin, will this indirect anti-microbial action be present.

The F4 and F7 films resulted not anti-microbial towards *S. aureus* and *Enterobacter* spp. by the agar diffusion test. The lack of antimicrobial activity observed could be attributable to a low density of CNs distributed along the surface of the samples. Li et al. [64] highlighted the importance of the CNs surface chemistry in their antimicrobial performance when loaded to a film making solution, confirming that partially deacetylated CNs exhibited superior antimicrobial activity despite the amount loaded when compared to non-deacetylated CNs. This could be explained by the increase in the number of amino groups being exposed on the chitin backbone, when a deacetylation process is involved [65]. 

On the other hand, Foster and Butt [66] reported an apparent lack of antimicrobial activity of thin chitosan films, with a 2% of CNs loaded, when tested against Gram positive and negative bacteria. The authors affirmed that even though chitosan solutions have demonstrated strong bactericidal activity against a wide range of bacteria, there is a loss of this beneficial property in thin films cast from the same solutions. Other authors reported antimicrobial activity of CNs when loaded to film making solutions in concentration range between 0.5% and 10% *w*/*w* [64,67], where agar diffusion tests had been performed on the solution, not on the films obtained from those solutions.

A relevant anti-microbial activity of CNs was observed for boards coated with CNs water suspension and this effect was demonstrated on fresh pasta [7]. In this case, the humidity and the hygroscopic behavior of cellulose granted the necessary condition for CNs to be active towards bacteria, discouraging their proliferation.

In agreement with the previous literature survey, the anti-microbial effects of CN and Chitosan are currently still under debate because of the different methodologies of applications. In our case, as the CNs are dispersed in the film by extrusion, the CNs are not reasonably exposed on the surface but embedded in the polymer bulk. Hence, the direct anti-microbial activity of the film is not significant, unless the concentration of CNs on the film surface is increased.

## 5. Conclusions

Nanobiocomposites suitable for preparing films by flat die extrusion were prepared based on plasticized poly(lactic acid) (PLA), poly(butylene succinate-co-adipate) (PBSA) and Chitin nanofibrils as functional filler. Chitin nanofibrils (CNs) were well dispersed thanks to the preparation of a pre-nanocomposites containing poly(ethylene glycol).

Thanks to the use of a melt strength enhancer (Plastistrength) and calcium carbonate the processability and thermal properties of bionanocomposites films containing CNs could be tuned in a wide range. The films resulted flexible and highly resistant.

The addition of CNs in the presence of starch proved not advantageous because of an extensive chain scission resulting in very low values of melt viscosity, making the material not suitable to be processed by flat die extrusion.

The films containing CNs or CNs and calcium carbonate were proven biocompatible and able to stimulate the production of cells defensins, acting as indirect anti-microbials.

Tests made with *Staphylococcus aureus* and *Enterobacter* spp. (Gram positive and negative respectively) by the qualitative agar diffusion test did not show any anti-microbial activity. The results can be explained considering that the CNs are present in the material bulk and not on the surface. Hence the direct anti-microbial activity, linked to the direct contact of the CNs with the bacteria cells, should be optimized.

The work suggests that for a compatible film with a mild indirect anti-microbial activity due to the defensins produced by skin cells, the incorporation of CNs in the film can be a good strategy. However, if a direct anti-microbial activity is required, it is necessary to increase the concentration of CNs on the film surface. Hence, developing coatings containing chitin nanofibrils can be an interesting perspective for future research regarding bioplastic films with a direct anti-microbial activity.

## Figures and Tables

**Table 1:** Primers sequences and Real-Time conditions.

Gene	Primer Sequence	Conditions	Size (bp)
**IL-1 α**	5′-CATGTCAAATTTCACTGCTTCATCC-3′ 5′-GTCTCTGAATCAGAAATCCTTCTATC-3′	5 s at 95 °C, 8 s at 55 °C, 17 s at 72 °C for 45 cycles	421
**IL-1 β**	5′-GCATCCAGCTACGAATCTCC-3′ 5′-CCACATTCAGCACAGGACTC-3′	5 s at 95 °C, 14 s at 58 °C, 28 s at 72 °C for 40 cycles	708
**TNF-α**	5′-CAGAGGGAAGAGTTCCCCAG-3′ 5′-CCTTGGTCTGGTAGGAGACG-3′	5 s at 95 °C, 6 s at 57 °C, 13 s at 72 °C for 40 cycles	324
**IL-6**	5′-ATGAACTCCTTCTCCACAAGCGC-3′ 5′-GAAGAGCCCTCAGGCTGGACTG-3′	5 s at 95 °C, 13 s at 56 °C, 25′ s at 72 °C for 40 cycles	628
**IL-8**	5-ATGACTTCCAAGCTGGCCGTG-3′ 5-TGAATTCTCAGCCCTCTTCAAAAACTTCTC-3′	5 s at 94 °C, 6 s at 55 °C, 12 s at 72 °C for 40 cycles	297
**TGF-β**	5′-CCGACTACTACGCCAAGGAGGTCAC-3′ 5′-AGGCCGGTTCATGCCATGAATGGTG-3′	5 s at 94 °C, 9 s at 60 °C, 18 s at 72 °C for 40 cycles	439
**HBD-2**	5′-GGATCCATGGGTATAGGCGATCCTGTTA-3′ 5′-AAGCTTCTCTGATGAGGGAGCCCTTTCT-3′	5 s at 94 °C, 6 s at 63 °C, 10 s at 72 °C for 50 cycles 5 s at 94 °C, 6 s at 63 °C, 10 s at 72 °C for 50 cycles	198

**Table 2:** Results of SEM and BET analysis related to chitin nanofibrils.

Chitin Average Length (nm)	Chitin Average Width (nm)	Average Fibrils Volume (cm^3^)	Average Mass of Each Fibril (g)	Number of Chitin Fibrils per Gram	Experimental BET (m^2^/g)	Theoretical BET (m^2^/g)
11,300	300	1.695 ×·10^−14^	2.4·×·10^−14^	4.15·×·10^13^	39.14	286.18

**Table 3:** PLA/PBS blends name and compositions.

Blends	PLA (wt.%)	PBS (wt.%)	ATBC (wt.%)	PS (wt.%)	PEG (%wt)	NC (wt.%)	Starch (wt.%)	Calcium Carbonate (wt.%)
F1	63	17	20	-	-	-	-	-
F2	62	16	20	2	-	-	-	-
F3	62	16	18	-	2 (PEG 6000)	2	-	-
F4	61	15	18	2	2 (PEG 6000)	2	-	-
F5	59	16	18	-	2 (PEG 400)	2	3	-
F6	58	15	18	2	2 (PEG 400)	2	3	-
F7	57.5	14.5	15	2	2 (PEG 6000)	2	-	7

**Table 4:** Torque, MVR and MFR values of the prepared blends.

Blends	Torque (N∙cm)	MVR (cm^3^/10 min)	MFR (g/10 min)
F1	67.8 ± 5.4	22.5 ± 2.0	23.6 ± 2.1
F2	72.8 ± 6.0	11.8 ± 0.9	12.4 ± 0.9
F3	60.0 ± 4.1	16.9 ± 1.5	18.4 ± 1.3
F4	63.3 ± 3.6	11.8 ± 1.1	13.1 ± 1.3
F5	42.0 ± 4.1	40.2 ± 10.5	43.5 ± 11.4
F6	38.6 ± 2.2	50.7 ± 6.0	55.0 ± 6.5
F7	73.5 ± 5.2	10.0 ± 1.3	11.4 ± 1.5

**Table 5:** Tensile properties of the films obtained from each blend.

Blends	Stress at Break σ_b_ (MPa)	Elongation at Break ε_b_ (%)	Stress at Yielding σ_y_ (MPa)
F1	31.8 ± 1.4	572.7 ± 20.7	-
F2	33.0 ± 1.2	554.2 ± 12.3	10.2 ± 0.7
F3	25.5 ± 1.2	455.2 ± 16.4	8.3 ± 1.5
F4	25.5 ± 1.3	421.9 ± 25.1	11.6 ± 0.7
F5	21.3 ± 3.1	398.7 ± 49.2	-
F6	19.7 ± 1.5	381.2 ± 27.5	-
F7	25.5 ± 1.0	400.1 ± 21.9	10.8 ± 1.8

**Table 6:** Results of differential scanning calorimetry analysis (first heating).

Blends	T_g_ (°C)	T_C_ (°C)	ΔH_C_ (J/g)	T_m_ (°C)	ΔH_m_ (J/g)	X_C_ %
F1	43.64	~96	3.05	143.74	18.84	27
F2	36.29	86.71	12.97	145.58	19.94	12
F4	43.90	87.52	19.31	145.91	20.57	2
F7	43.55	85.72	11.97	145.46	18.82	13

**Table 7:** Results of differential scanning calorimetry analysis (second heating).

Blends	T_g_ (°C)	T_m,PBS_ (°C)	ΔH_m,PBS_ (J/g)	T_C,PLA_ (°C)	ΔH_C,PLA_ (J/g)	T_m,PLA_ (°C)	ΔH_m,PLA_ (J/g)	X_C_ %
F1	26.96	79.63	3.74	88.21	11.49	144.27	22.62	19
F2	33.54	83.40	6.83	96.72	16.90	145.40	20.54	6
F4	34.75	84.87	5.81	96.53	20.06	147.24	22.87	5
F7	30.24	83.72	6.68	94.87	15.00	146.01	20.07	9

**Table 8:** % of ABred in keratinocytes treated for 24 h with F4 and F7.

Sample	% AB_RED_
F4	150
F14	137
